# Study on Preprocessing Methods for Ultrasonic Signals from Internal Defects in Rolls

**DOI:** 10.3390/s26123769

**Published:** 2026-06-12

**Authors:** Baotong Chen, Xiaolong Hu, Xuguo Yan, Shiyang Zhou

**Affiliations:** 1 Key Laboratory of Metallurgical Equipment and Control Technology, Wuhan University of Science and Technology, Ministry of Education, Wuhan 430081, China; mecheney@wust.edu.cn; 2 Hubei Key Laboratory of Mechanical Transmission and Manufacturing Engineering, Wuhan University of Science and Technology, Wuhan 430081, China; dragonhu283@gmail.com (X.H.); 15623061079@163.com (S.Z.); 3 State Key Laboratory of Intelligent Manufacturing Equipment and Technology, Huazhong University of Science and Technology, Wuhan 430074, China

**Keywords:** roll, internal defect, feature extraction, ultrasonic testing

## Abstract

Accurate detection of internal defects in rolls is crucial for industrial safety and product quality. Ultrasonic testing is a mainstream non-destructive method widely used for this purpose. However, in practice, ultrasonic echo signals often suffer from background clutter. When defects are located near the surface, weak defect echoes tend to couple with surface echoes, making signal extraction difficult and reducing the accuracy of subsequent feature extraction and classification. This paper proposes a novel ultrasonic signal preprocessing method aimed at improving the performance of subsequent defect identification models. The method first acquires ultrasonic signals from defect regions and background clutter reference signals from defect-free regions using a digital ultrasonic flaw detector. An improved median filter is then applied to remove spike interference and boundary outliers. On this basis, a multi-stage FIR (finite impulse response) filter is constructed, and particle swarm optimization is employed to adaptively optimize filter parameters, achieving an accurate estimation of background clutter. Finally, the clutter-suppressed defect signal is obtained through signal subtraction. Experimental results on a dataset of 5000 samples (2500 defective, 2500 non-defective) containing cylindrical artificial defects (diameter 8 mm, length 30 mm) demonstrate that using a CNN classifier with the same feature extraction and classification model, the signals preprocessed by the proposed method outperform traditional median filtering and wavelet denoising methods. The defect identification accuracy is improved by approximately 38 percentage points compared to median filtering and 20 percentage points compared to wavelet denoising, while also achieving a high recall rate, validating the effectiveness of the proposed method in enhancing roll internal defect detection.

## 1. Introduction

Work rolls, as core load-bearing components in industrial sectors such as steel rolling and pipe processing, have their service life directly determined by internal defects (e.g., cracks, porosity, and looseness), which are also key factors triggering roll failure and threatening production safety [1]. Roll failure may not only cause prolonged production line shutdowns resulting in substantial economic losses, but may also lead to safety accidents due to sudden component fracture. Therefore, the accurate detection of internal defects is essential.

In current industrial scenarios, ultrasonic testing [2] has become the mainstream method for the non-destructive testing of roll internal defects owing to its wide detection range, good directionality, and high sensitivity. Its basic principle involves analyzing the reflection and refraction variations of ultrasonic waves propagating within the roll [3], thereby assessing material properties and internal structural anomalies to identify surface and internal defects. However, the quality of ultrasonic echo signals in practical inspection is highly susceptible to multiple interfering factors. Echo signals contain not only surface echoes [4], bottom echoes, and defect echoes, but also substantial noise interference [5]. Particularly when defects are located near the roll surface, defect echoes tend to undergo nonlinear coupling with surface echoes [6], causing weak defect echoes to be masked and severely restricting defect extraction accuracy. In addition, unstable coupling between the probe and roll surface during signal acquisition introduces spike pulse noise [7] (i.e., “burrs”), further increasing clutter estimation bias and interfering with subsequent processing.

Many filtering methods have been proposed, but each has significant limitations. Frequency filtering methods [8] can only remove noise within specific frequency bands and exhibit poor adaptability to noise with substantially different frequency characteristics across different inspection scenarios. The smoothing effect of mean filtering [9] relies heavily on the local data interval size, making it difficult to simultaneously balance signal sensitivity and noise immunity. Although traditional median filtering can suppress occasional pulse interference, it does not address the “boundary outlier” problem in roll ultrasonic signals and cannot effectively distinguish coupling anomaly points near the surface echo from genuine defect signals. Wiener filtering [10] employs fixed parameters and is only suitable for stationary random signals, making it difficult to adapt to the non-stationary characteristics of roll ultrasonic signals. Improved methods also have shortcomings. Wu et al.’s adaptive time-frequency filtering [11] requires a reference spectral model that depends on difficult-to-obtain prior parameters. Yang et al.’s variational mode decomposition [12] requires presetting decomposition layers and penalty factors, and its performance is sensitive to initial values. Fang et al.’s denoising method [13] depends on high-quality mask generation and may amplify random fluctuations in non-defect regions. None of the above approaches can simultaneously address two key challenges existing in roll ultrasonic signals, including spike pulse noise induced by unstable probe coupling and time-varying background clutter that nonlinearly couples with weak defect echoes.

Focusing on improvements to median filtering, researchers have explored various dimensions including structural design, threshold strategies, and optimization-based fusion. Nalli et al. [14] cascaded an adaptive filter with a median-algorithm-optimized secondary filter, improving the image processing quality; Lin et al. [15] realized reconfigurable one-dimensional median filtering through heterogeneous median unit cascading, introducing predefined thresholds to maintain operational rate stability while reducing hardware costs; Appiah et al. [16] developed two improved approximate median filtering algorithms based on dynamic programming, balancing operational efficiency and output quality; Ai [17] integrated wavelet-regularized sharpening median filtering with a preprocessing model, combining the advantages of noise suppression, edge enhancement, and frequency-domain restoration to significantly improve the peak signal-to-noise ratio and structural similarity; Saleh et al. [18] proposed a median filtering framework based on the crow optimization algorithm, using iterative search for the global optimal median to replace noise pixels and improving denoising efficiency while maintaining image structural integrity. The above improved strategies are mostly oriented toward two-dimensional image processing and lack specificity for probe coupling anomaly points and complex background clutter coupling problems in one-dimensional ultrasonic signals. Moreover, some methods involve complex parameter settings and limited adaptability to noise variations, making them difficult to directly satisfy the requirements of roll ultrasonic testing. Therefore, it is necessary to explore preprocessing methods better suited to the non-stationary characteristics of ultrasonic signals.

To fill the gap, this paper proposes a novel preprocessing method that combines an improved median filter with a PSO-optimized multi-stage FIR filter. The improved median filter introduces a dynamic outlier correction mechanism that replaces abnormally high amplitudes (caused by unstable coupling) with three times the median value, effectively suppressing spike pulses and boundary outliers. The PSO-optimized multi-stage FIR filter [19] adaptively estimates the background clutter from a defect-free reference signal and subtracts it from the defect signal, achieving clean defect echo extraction. This combination is superior because the two stages address complementary interference: the first removes impulsive noise, while the second cancels non-stationary clutter—a synergy that none of the existing methods provide. Experimental results demonstrate that when using the same CNN classifier, signals preprocessed by the proposed method achieve higher defect identification accuracy and faster convergence than traditional filtering methods, providing a high-quality foundation for deep learning-based automatic defect identification.

The remainder of this paper is organized as follows. Section 2 elaborates on the technical approach of the proposed method, detailing the window parameter optimization and boundary outlier correction mechanism of the improved median filter, the construction of the multi-stage FIR filter, and the PSO-based adaptive parameter solving strategy. Section 3 describes the experimental platform setup, preparation of artificial defect specimens, and the evaluation framework, covering data acquisition schemes, the software environment, and the multi-level comparative experimental design. Section 4 presents the experimental results and analysis from three aspects—window parameter optimization, filter order determination, intelligent algorithm comparison, and preprocessing method performance comparison—and validates the engineering feasibility of the method in practical industrial scenarios. Section 5 summarizes the work and discusses future research directions.

## 2. Proposed Method

**Figure 1:**
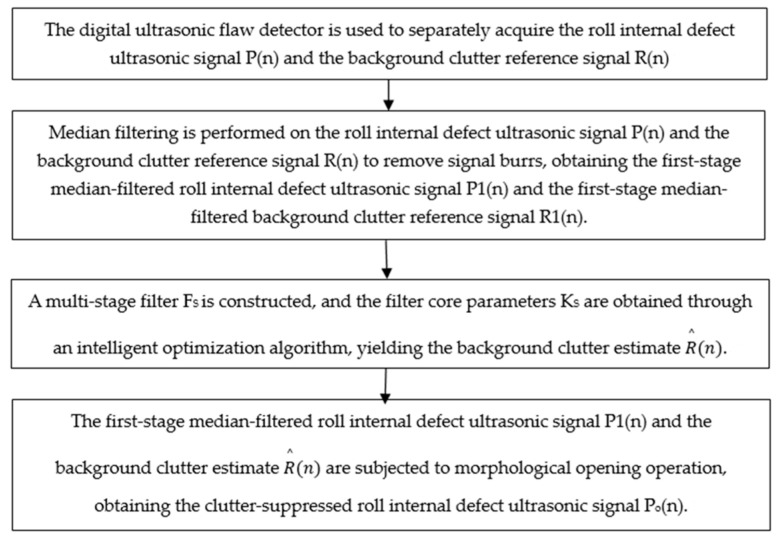
Flowchart of the ultrasonic signal filtering method for roll internal defects.

The technical route of the proposed method is illustrated in Figure 1.

In roll ultrasonic testing, the original echo signals typically contain substantial spike pulse noise (burrs) and boundary outliers introduced near the surface echo due to unstable probe coupling. These interferences not only degrade signal quality but also severely affect the accuracy of subsequent feature extraction and classification tasks.

Traditional median filtering achieves noise suppression by sorting the sampling points within a sliding window and selecting the median value. Its basic expression is given by:

(1) $$X_{n}=med\left(x_{n-m},x_{n-m+1},x_{n},\cdots ,x_{n+m}\right)$$

where med(⋅) denotes the median function, m is the window radius, and 2m + 1 is the window width. However, this traditional approach has the following limitations: it cannot effectively distinguish coupling anomaly points near the surface echo from genuine defect signals; a fixed window size struggles to balance noise suppression and feature preservation; and it performs poorly in suppressing boundary outliers with large amplitudes.

To address these issues, an improved median filtering algorithm is proposed in this paper. Taking window radius as the core optimization parameter, the optimal value was determined through systematic comparison of the variation trends in signal-to-noise ratio (SNR) and root mean square error (RMSE) under different window sizes. A window radius of 12 was found to achieve the best balance between pulse noise suppression and defect echo detail preservation.

In terms of the outlier correction mechanism, a dynamic detection and correction procedure was introduced. For signal elements at non-central positions within the filtering window, if their amplitude exceeds three times the window median value, they are identified as abnormally high-amplitude points caused by unstable probe coupling and are subsequently corrected to three times the median value. Theoretically, this method achieves good suppression of boundary outliers and prevents them from interfering with subsequent defect extraction. This threshold is determined as an empirical value based on statistical theory and the physical characteristics of ultrasonic signals. In practical application, the digital ultrasonic flaw detector is first used to separately acquire the roll ultrasonic signal P(n) containing internal defects and the background clutter reference signal R(n) measured in defect-free regions. The improved median filtering algorithm is then applied to both signals as the first-stage preprocessing to effectively suppress spike pulse interference.

Primary preprocessing suppresses impulsive noise well, yet clutter-defect echo nonlinear coupling restricts detection accuracy improvement. This type of clutter consists of multiple superimposed components, including equipment noise, material scattering, and interface reflections, with characteristics that vary dynamically with operating conditions. Fixed-parameter filters struggle to achieve deep suppression while fully preserving defect features. To address this, a multi-stage FIR filter is introduced as the core of the second-stage processing, generating a high-precision clutter estimate through intelligent fitting of the preprocessed background clutter reference signal.

The multi-stage FIR filter F_S_ constructed in this paper has an order d, with its corresponding filter weight vector denoted as $\omega =\left[\omega_{0},\omega_{1},...,\omega_{d-1}\right]$, where $\omega_{0}$ to $\omega_{d-1}$ are the filter coefficients to be optimized. The filter output, i.e., the background clutter estimate R^(n), is determined by the convolution of the weight vector with the input signal (the background clutter first-stage median-filtered reference signal R_1_(n)). Its discrete-time expression is:

(2) R^(n)=∑k=0d−1ωk·R1n−k

To simplify the expression and highlight its vector operation form, the above equation can be rewritten as:

(3) R^(n)=ω·[r1(n),r1(n−1),⋯,r1(n−d+1)]T

Through preliminary experimental comparison, the filter order was determined to be 5, yielding the weight vector:

(4) $$\omega =\left[\omega_{0},\omega_{1},\omega_{2},\omega_{3},\omega_{4}\right]$$

The background clutter estimate R^(n) can then be specified as:

(5) R^(n)=ω·[r1(n),r1(n−1),r1(n−2),r1(n−3),r1(n−4)]T

Conventional methods such as least squares estimation require matrix inversion and are sensitive to data characteristics, leading to insufficient stability under non-stationary clutter conditions. To overcome this limitation, particle swarm optimization (PSO) is introduced in this paper, transforming the filter weight vector determination into a global optimization problem. The specific solution steps and core iterative formulas of the algorithm are as follows. First, a particle population is initialized, where each particle represents a set of candidate filter weight vectors $x_{i}$ with a randomly assigned velocity $v_{i}$. In each iteration, particles update their velocities and positions based on their individual historical best position ${\;pbest}_{i}$ (individual optimal filter weights) and the global best position *gbest* (global optimal filter weights) of the entire population. The core iterative formulas are:

(6) $$v_{i}\left(t+1\right)=\omega v_{i}\left(t\right)+c_{1}r_{1}\left(pbest_{i}-x_{i}\left(t\right)\right)+c_{2}r_{2}\left(gbest_{i}-x_{i}\left(t\right)\right)$$

(7) $$x_{i}\left(t+1\right)=x_{i}\left(t\right)+v_{i}\left(t+1\right)$$

where ω is the inertia weight, $c_{1},c_{2}$ are learning factors, $r_{1},\;{\;r}_{2}$ are random numbers in the interval $\left[0,1\right]$, and t is the current iteration number. Subsequently, the objective function J value for each particle is calculated, and the individual best and global best solutions are updated. The iterative process continues until the convergence condition is satisfied, and the final global best particle position is output as the optimal filter weight vector.

The objective function of the intelligent optimization algorithm is defined as:

(8) $$J=\sum_{i=0}^{n}e{\left(i\right)}^{2}=\sum_{i=0}^{n}{\left(R_{1}(i)-\omega {\cdot [r_{1(i)},r_{1(i-1)},r_{1(i-2)},r_{1(i-3)},r_{1(i-4)}]}^{T}\right)}^{2}$$

A smaller value of the objective function J indicates more accurate background clutter estimation and more thorough defect signal separation.

To quantify the deviation between P_1_(n) and the background clutter estimate R^(n) output by the multi-stage filter, the error signal e(n) is established as:

(9) en=P1n−R^(n)

Substituting the expression for R^(n) from Equation (5) yields:

(10) $$e(n)\;=P_{1}(n)-\omega \cdot {\left[r_{1n},r_{1(n-1)},r_{1(n-2)},r_{1(n-3)},r_{1(n-4)}\right]}^{T}$$

The optimized optimal weight vector and the first-stage median-filtered background clutter reference signal $R_{1}(n)$ are then substituted into the filter expression to obtain the background clutter estimate R^(n). Finally, the clutter-suppressed roll internal defect ultrasonic signal Po(n) is obtained by subtracting this estimate from the first-stage median-filtered defect signal $P_{1}\left(n\right)$.

## 3. Experimental Design

**Figure 2:**
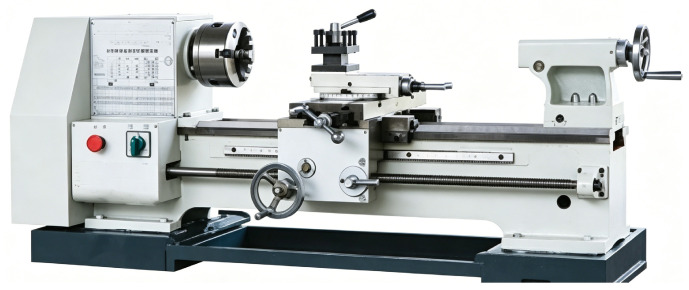
Roll ultrasonic testing experimental platform modified from a universal lathe.

To validate the effectiveness of the proposed method, a comprehensive experimental verification framework was established, encompassing signal acquisition, software processing, and performance evaluation. The experimental data were acquired from a roll ultrasonic testing experimental platform modified from a universal lathe. This platform consists of three subsystems—a mechanical system, a detection system, and a data processing system (as illustrated in Figure 2). The mechanical system comprises a cast iron testing bench, a roll fixture, and a probe movement mechanism driven by a stepper motor, enabling precise movement in both circumferential and axial directions with an adjustable step size ranging from 0.1 mm to 10 mm. The detection system includes a KY-CT350B digital ultrasonic flaw detector (KY-CT350B digital ultrasonic flaw detector (Kuyang Precision Measuring Instruments (Shanghai) Co., Ltd., Shanghai, China)), a 2.5 MHz straight-beam probe with a diameter of 20 mm, and an industrial-grade ultrasonic couplant. The data processing system consists of a PC and an ultrasonic flaw detection data management system, supporting real-time waveform display, data storage, and report generation.

**Figure 3:**
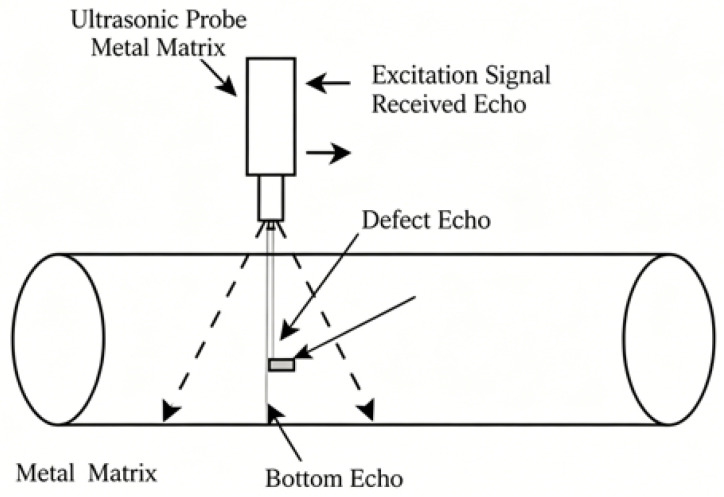
Schematic diagram of the ultrasonic inspection principle for roll internal defects.

**Figure 4:**
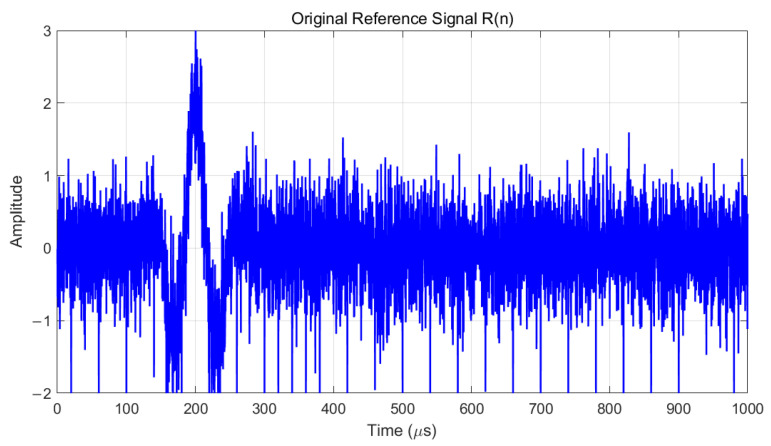
Original background signal R(n).

**Figure 5:**
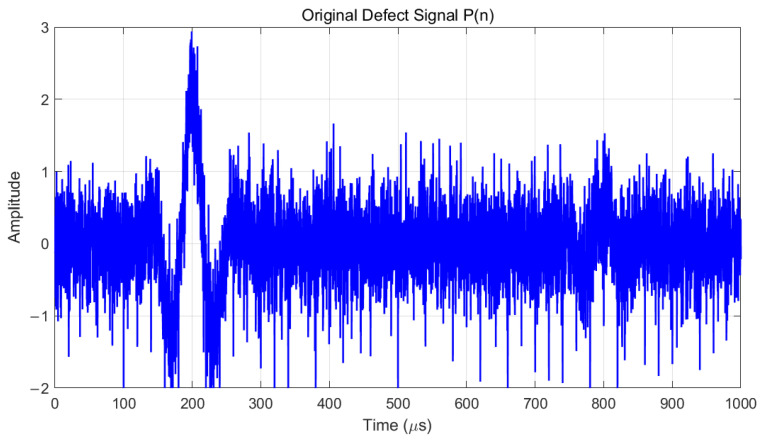
Original defect signal P(n).

The experimental specimen was a #45 steel roll with a length of 400 mm and a diameter of 130 mm. The material properties include an elastic modulus of 206 GPa, a Poisson’s ratio of 0.3, and a density of 7850 kg/m^3^, consistent with the dimensions of actual small- to medium-sized rolls. Artificial defects were fabricated inside the specimen using CNC (computer numerical control) drilling for subsequent method validation. The artificial defect was a cylindrical hole with a diameter of 8 mm and a length of 30 mm, fabricated by CNC drilling. Based on preliminary experiments, when a defect is placed too close to the surface (e.g., within 1–2 mm), the defect echo couples severely with the surface echo, interfering with the validation of the proposed method. Therefore, to avoid near-surface coupling effects, the defect was placed at a depth of 10 mm from the roll surface, which is far beyond the near-surface blind zone of the ultrasonic probe. This ensures that the reference signal and the defect signal are clearly separable. During the signal acquisition process, the roll specimen was first fixed onto the fixture, and the probe was adjusted to be perpendicular to the roll surface. A schematic diagram of the ultrasonic inspection principle is shown in Figure 3. The flaw detector was connected to the PC and set to online mode, with the sound velocity configured at 5900 m/s and the detection range covering 0 to 130 mm. The couplant was uniformly applied to the roll surface to ensure good acoustic coupling, and the probe movement mechanism was activated to acquire echo data along the predetermined scanning path. During the experiment, both the ultrasonic signal P(n) from regions containing internal defects and the background clutter reference signal R(n) from defect-free regions were simultaneously acquired. The defect-free regions were verified by performing a high-resolution C-scan over the candidate area before signal acquisition; no echo exceeding the noise floor was observed. For each specimen, ten reference measurements were taken at different positions to ensure representativeness, and the averaged reference signal was used as R(n). As shown in Figure 4 and Figure 5,the echo data were stored in file format and subsequently imported into the PC. All signal processing and algorithm implementation, including improved median filtering, multi-stage FIR filter construction, PSO parameter optimization, and subsequent performance evaluation, were carried out in the MATLAB R2023b environment.

To systematically evaluate the effectiveness of the proposed method, the experimental design was structured across three levels. At the window parameter optimization level, a controlled variable approach was adopted to compare the variation trends of SNR and RMSE as the window radius increased from 7 to 20, analyzing the marginal benefit of performance improvement to determine the optimal window size. At the filter order determination level, the optimal FIR filter order was determined by testing d = 2 to 10 using the least-squares method, balancing the objective function J and the AIC (Akaike information criterion). At the intelligent optimization algorithm comparison level, the convergence speed and solution accuracy of the PSO algorithm, genetic algorithm (GA), and simulated annealing (SA) algorithm were compared on the same dataset, with the parameter configurations of each algorithm strictly following a unified setup to ensure fairness. At the preprocessing method performance comparison level, the proposed method was compared with three traditional preprocessing methods—median filtering, Wiener filtering, and wavelet denoising. The signals processed by these four methods were separately fed into the same CNN (convolutional neural network) classifier, and multiple metrics, including accuracy, precision, recall, F1-score, and AUC (area under the curve), were used to comprehensively assess the practical application performance of each preprocessing method, thereby thoroughly validating the superiority of the proposed method in background clutter suppression and defect feature preservation. To ensure consistent ultrasonic coupling, the roll surface was ground prior to inspection to eliminate the influence of surface roughness on the echo signals.

## 4. Results and Discussion

To validate the rationality of the parameter selection in each step of the proposed method and the overall preprocessing effectiveness, this section presents the experimental results and analysis in three progressive aspects: optimization of the improved median filter window, comparison of multi-stage filter parameter solving algorithms, and the impact of different preprocessing methods on defect classification performance.

### 4.1. Window Parameter Experiment

**Figure 6:**
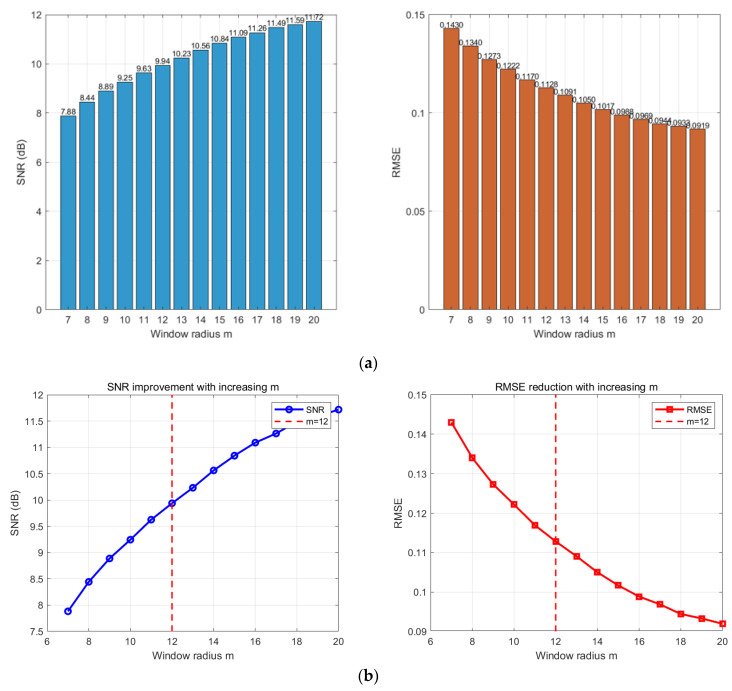
(**a**) Bar chart of SNR and RMSE under different window radii; (**b**) SNR and RMSE versus window radius m.

For the selection of the window radius in the improved median filter, the filtering performance was systematically examined using SNR and RMSE as evaluation metrics as the window radius increased from 7 to 20, as presented in Table 1 and Figure 6.

As the window radius increased from 7 to 20, the SNR of the filtered signal continuously improved from 7.88 dB to 11.72 dB, and the RMSE monotonically decreased from 0.1430 to 0.0919, indicating that a larger window radius can effectively suppress noise and reduce the deviation from the ideal signal. However, the incremental gains between adjacent radii showed a gradually decreasing trend. When the radius increased from 7 to 10 (as shown in Table 2), the SNR increments ranged from 0.36 to 0.56 dB, and the RMSE reductions were between 0.005 and 0.009. After the radius exceeded 12, the SNR increments generally fell below 0.30 dB, and in the later stage (m ≥ 16), further dropped below 0.25 dB, with RMSE reductions narrowing to within 0.003. Although no positive RMSE increment (which would indicate over-smoothing) was observed within the tested range, an excessively large window radius inevitably increases the risk of over-smoothing, potentially causing the loss of fine details in weak defect echoes. Considering both marginal benefit and signal fidelity, the window radius of 12 (corresponding to SNR = 9.94 dB and RMSE = 0.1128) lies exactly in the transition region where performance improvement shifts from significant to marginal. Therefore, it was determined as the optimal window radius for the improved median filter.

**Figure 7:**
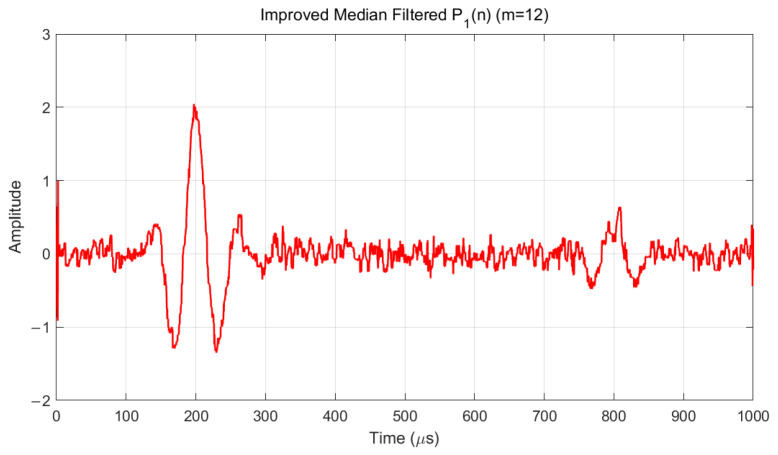
First-stage median-filtered roll internal defect ultrasonic signal P1(n).

**Figure 8:**
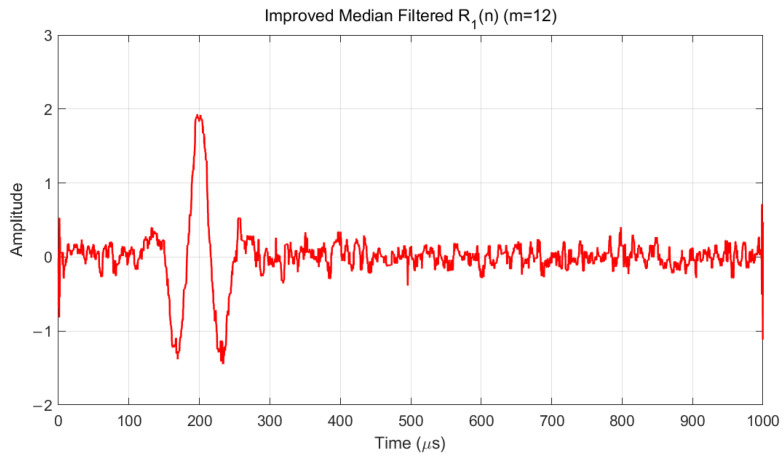
First-stage median-filtered background clutter signal R1(n).

The first-stage median-filtered defect signal P1(n) and background clutter reference signal R1(n) obtained after this processing are shown in Figure 7 and Figure 8, respectively, where the spike pulse noise has been effectively suppressed.

### 4.2. Filter Order Experiment

The order d of the multi-stage FIR filter directly affects the accuracy of background clutter estimation and the model complexity. To systematically determine the optimal order, the least-squares method was used to solve the filter coefficients for different orders, which obtains the optimal parameter estimates by minimizing the sum of squared errors. The objective function: $J=\sum_{i=0}^{N}e{\left(i\right)}^{2}.$ served as the metric for fitting accuracy, and the Akaike information criterion (AIC) was also computed to balance model complexity and goodness-of-fit. Typically, the optimal order is indicated by a significant decrease in J followed by a plateau, while the AIC value decreases to its minimum and levels off; the order satisfying such characteristics is selected as the optimal filter order. Experiments were carried out using the same ultrasonic signal model as in the window parameter optimization, testing orders d = 2 to 10. The results are listed in Table 3.

As shown in Table 3, the objective function J decreased monotonically with increasing order d, indicating that higher-order filters achieved a better fit of the background clutter. However, the marginal benefit analysis revealed that when the order increased from 2 to 5, the relative reduction in J was about 1.4–1.5%; when the order increased from 5 to 6, the reduction dropped sharply to 0.65%. Afterward, the reduction fluctuated at a relatively low level (0.4–1.3%). Although the AIC value continued to decrease, its decreasing rate also slowed down gradually. Considering both the marginal improvement in fitting accuracy and the model complexity, the order of 5 lies at the turning point where the performance gain changes from significant to marginal—the J value had already reached a low level with limited subsequent gain, and the improvement in AIC also diminished significantly. Therefore, d = 5 was selected as the optimal order for the multi-stage FIR filter. It should be noted that this optimization was performed only for the signal model under the current inspection conditions. For different defect types, material properties, or signal-to-noise ratios, the optimal order may vary. Developing an adaptive order selection strategy for varying operating conditions will be an important direction for future research.

### 4.3. Intelligent Optimization Algorithm Comparison Experiment

To determine the optimal weight vector for the multi-stage FIR filter, the convergence performance of three intelligent algorithms—particle swarm optimization (PSO), genetic algorithm (GA), and simulated annealing (SA)—in minimizing the objective function J was compared. The core mechanisms by which each algorithm solves the filter weight vector are described as follows.

The core iterative formulas of the PSO algorithm can be found in Section 2.

The GA algorithm simulates the mechanisms of natural selection and genetic evolution, progressively optimizing the filter weight vector through selection, crossover, and mutation operations on individuals in the population. The selection operation adopts a roulette wheel strategy, where the probability of individual *i* being selected is proportional to its fitness value $f_{i}.$

(11) $$p_{i}=\frac{f_{i}}{\sum f_{i}}$$

The crossover operation performs real-coded arithmetic crossover on the selected parent individuals with crossover probability $p_{c}$, generating offspring:

(12) $$x_{1}^{\prime }=\alpha x_{1}+\left(1-\alpha \right)x_{2}$$

(13) $$x_{2}^{\prime }=\left(1-\alpha \right)x_{1}+\alpha x_{2\;}$$

where $\alpha \in \left[0,\;1\right]$ is a random weight coefficient. The mutation operation applies random perturbations to individual gene positions with mutation probability $p_{m}$,

(14) $$x_{i}=x_{i}+\delta$$

where δ is a small random variable following a uniform distribution, thereby maintaining population diversity and avoiding premature convergence.

The SA algorithm draws on the statistical physics principles of the solid annealing process, employing a probabilistic acceptance mechanism to escape local optima. The algorithm starts from an initial temperature $T_{0}\;$ and a random initial solution. In each iteration, random perturbations are applied to the current solution to generate a neighborhood candidate solution, with the acceptance criterion based on the objective function difference $△J=J_{new}-J_{old}$. If △J<0, the new solution is accepted; if △J≥0, the inferior solution is accepted with probability:

(15) $$P=exp\left(△J/T\right)$$

The temperature is then gradually decreased according to a cooling coefficient q,

(16) $$T_{k+1}=q\cdot T_{k}\;\left(0<q<1\right)$$

allowing the algorithm to thoroughly explore the solution space at high temperatures and tend toward convergence at low temperatures.

The parameter settings for each algorithm strictly followed the standard configuration presented in Table 4 to ensure the fairness of the comparative experiment. The convergence performance of the PSO algorithm is closely related to the inertia weight *w* and the learning factors $c_{1}$, $c_{2}$. The inertia weight balances global exploration and local exploitation; according to mainstream PSO studies, *w* is typically chosen within the range of 0.4 to 0.9. The learning factors control the extent to which a particle follows its own historical best and the population’s global best. Based on classical literature and numerous engineering applications, $c_{1}$ and $c_{2}$ are often set to around 2.0. In this paper, a small-scale comparative experiment was conducted within the above parameter ranges, using the convergence speed and the final value of the objective function as evaluation criteria. Consequently, the inertia weight was set to *w* = 0.8, the individual learning factor to $c_{1}=0.5$, and the global learning factor to $c_{2}=0.6$. This parameter set exhibited fast convergence and a low final objective function value in the experiments. The specific parameter configuration of the PSO algorithm is shown in Table 4. The crossover and mutation rates of the GA were set to 0.85 and 0.15, respectively. The initial temperature $T_{0}$ and cooling rate q of the simulated annealing algorithm directly affect the convergence speed and global search ability. According to classical studies, the initial temperature is usually set between 50 and 100, and the cooling rate between 0.8 and 0.99. In this paper, initial temperatures in the range of 50–100 were tested, and cooling rates from 0.85 to 0.95 were compared with respect to the final objective function value. The chosen parameters $T_{0}=$ 50 and q = 0.92 yielded stable optimal solutions within a reasonable number of iterations.

To ensure a fair comparison, all three algorithms were run under the same total number of objective function evaluations. The objective function $J=\sum {e\left(i\right)}^{2}$ is the core metric for evaluating algorithm performance, and its computational cost depends on the signal length and filter order. In this study, the total number of evaluations was set to 3000 for each algorithm. Specifically, PSO used 50 particles over 60 iterations (50 × 60 = 3000), GA used a population of 50 over 60 generations (50 × 60 = 3000), and SA used 3000 single-point iterations. Each algorithm was independently run 50 times to obtain statistically meaningful results. The detailed parameter settings are listed in Table 5.

**Figure 9:**
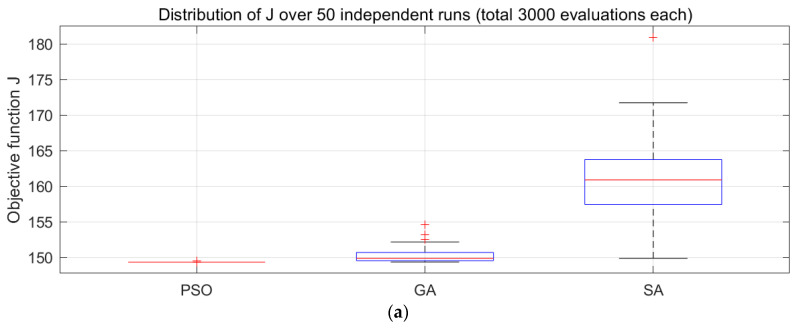

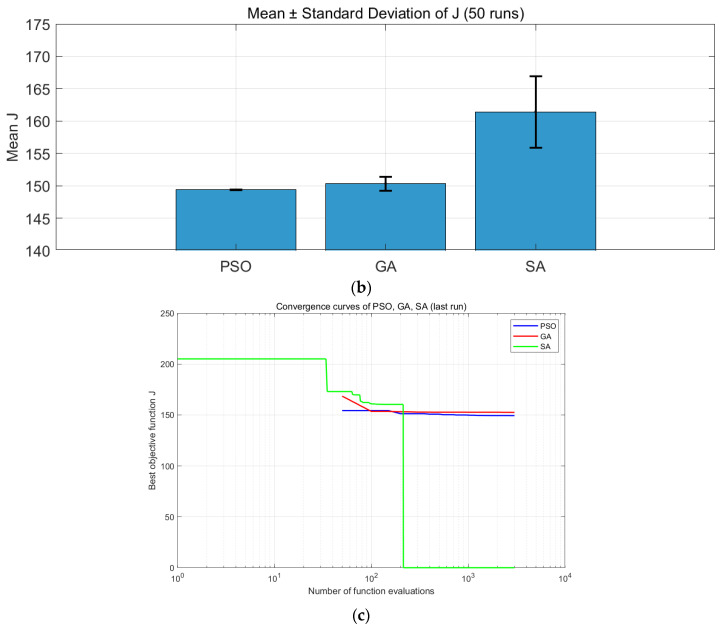
Algorithm performance comparison: (**a**) boxplot of J; (**b**) mean ± std of J; (**c**) convergence curves.

Figure 9 present the performance comparison. Over the 50 runs, PSO achieved a mean objective function of 149.366 with a standard deviation of 0.023; GA yielded 150.325 ± 1.091; and SA yielded 161.412 ± 5.519. Wilcoxon signed-rank tests indicate that the differences between PSO and GA and between PSO and SA are highly statistically significant $\left(p<10^{-9}\right)$, as shown in Table 6. Figure 9a,b (boxplot and bar chart with error bars) visually confirm the superiority and robustness of PSO.

It should be noted that recording the convergence history for every evaluation across all 50 runs would consume excessive memory and is unnecessary. Therefore, we only present the convergence curves of the last run (the 50th run) in Figure 9c as a representative example of the typical convergence behavior of each algorithm. From Figure 9c, it can be observed that PSO converged rapidly to a low objective function value within a relatively small number of evaluations, while SA exhibited the slowest convergence with significant fluctuations. Consequently, PSO was selected as the optimization method for the multi-stage FIR filter, yielding the optimal coefficient vector Ks = [0.9671, 0.0162, 0.0069, 0.0014, 0.0002].

**Figure 10:**
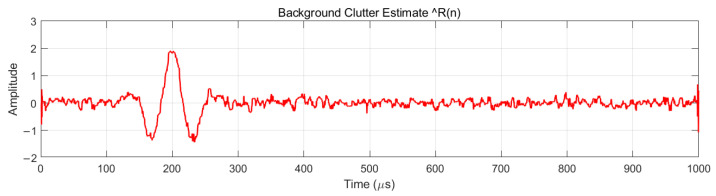
Background clutter estimate R^(n).

**Figure 11:**
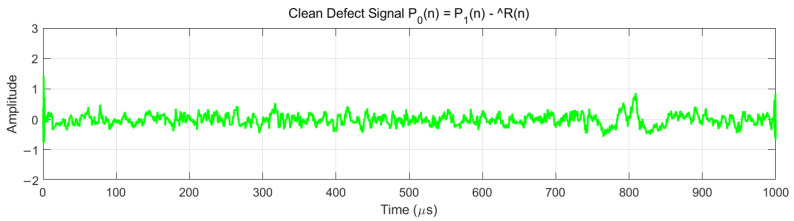
Clutter-suppressed roll internal defect ultrasonic signal Po(n).

Subtracting the background clutter estimate R^(n) (as shown in Figure 10) from the first-stage median-filtered roll internal defect ultrasonic signal P1(n) yields the clutter-suppressed roll internal defect ultrasonic signal Po(n), the final waveform of which is shown in Figure 11.

After processing with the improved median filter with a window radius of 12 and the PSO-optimized multi-stage FIR filter of order 5, the resulting Po(n) signal still contained minor local burrs with an amplitude of approximately ±0.5. Their sources can be attributed to the following factors. High-amplitude spike pulses in the original signal, although substantially attenuated by median filtering, may still leave residual weak fluctuations that manifest in the subsequent subtraction operation. The PSO algorithm minimizes the global mean square error as its objective and does not enforce strictly zero error at every sampling point, so small deviations at individual positions are an inherent characteristic of this method. Additionally, at the initial segment of the signal, the filter lacks sufficient delay samples and employs boundary replication processing, which may also introduce transient jumps. The amplitudes of these residual fluctuations are far lower than those of typical defect echoes, and the defect region maintains a clear waveform contour without significant distortion, indicating that they do not substantially affect subsequent identification.

### 4.4. Preprocessing Method Comparison Experiment

To ultimately evaluate the practical improvement in defect identification performance achieved by the proposed method, the PSO-optimized multi-stage FIR filter preprocessing method was compared with three traditional preprocessing methods, namely median filtering, Wiener filtering, and wavelet denoising. The basic principles of the three traditional methods are described below.

Median filtering is based on the theory of order statistics. It sorts the sampling values within a sliding window and replaces the original value at the window center with the median value, thereby suppressing pulse noise. The corresponding expression is shown in Equation (1). 

Wiener filtering is an optimal linear filter based on the minimum mean square error criterion, achieving separation of signal and noise in the frequency domain. Let the noisy signal be:

(17) $$y\left(n\right)=s\left(n\right)+v\left(n\right)$$

where $s\left(n\right)$ is the true signal and $v\left(n\right)$ is additive noise. The frequency-domain transfer function of the Wiener filter is:

(18) $$H\left(f\right)=\frac{P_{s}f}{P_{s}f+P_{v}f}$$

where $P_{s}f$ and $P_{v}f$ are the power spectral densities of the signal and noise, respectively.

Wavelet denoising exploits the multi-resolution analysis capability of the wavelet transform, decomposing the signal into sub-bands at different scales and then applying thresholding to the wavelet coefficients of each layer to separate the noise. First, the noisy signal undergoes discrete wavelet transform to obtain the wavelet coefficients $\omega_{j,k}$ at each scale. Then, soft-thresholding or hard-thresholding functions are applied. The expression for the soft-thresholding function is:

(19) $$\hat{\omega }=\left\{\begin{array}{cc} \;\;\;sgn\left(\omega_{j,k}\right)\left(\left|\omega_{j,k}\right|-\lambda \right), & \;\;\;\;\;\left|\omega_{j,k}\right|\geq \lambda \\ 0, & \;\;\;\;\;\left|\omega_{j,k}\right|<\lambda \;\;\;\;\; \end{array}\right.$$

where λ is the threshold parameter and sgn(⋅) is the sign function.

The experimental dataset consisted of 5000 ultrasonic signal samples, equally divided into 2500 defective and 2500 non-defective samples. The additive noise in the ultrasonic signals was modeled as zero-mean white Gaussian noise with a standard deviation determined by the signal-to-noise ratio (SNR). A 5-fold cross-validation scheme was adopted, where each fold used 80% of the data for training (including an internal validation set) and 20% for testing, resulting in five independent training-testing rounds. The mean and standard deviation of each performance metric were reported across the five folds. All random seeds were fixed to 42 to ensure reproducibility. The signals preprocessed by the four methods were separately fed into a CNN classifier with an identical structure for training and testing. The CNN model comprised three one-dimensional convolutional layers followed by three fully connected layers. The first convolutional layer contained 16 kernels of size 5 × 1 with stride 1 and “same” padding; the second convolutional layer contained 32 kernels of size 5 × 1 with stride 1 and “same” padding; the third convolutional layer contained 64 kernels of size 3 × 1 with stride 1 and “same” padding. Each convolutional layer was followed by a batch normalization layer (for accelerating convergence and providing regularization), a ReLU activation function, and a max-pooling layer of size 2 × 1 with stride 2 (which halves the length of the feature maps). Subsequently, three fully connected layers with 64, 32, and 2 neurons were used. Dropout layers with rates of 0.5 and 0.3 were added after the first two fully connected layers, respectively, to prevent overfitting. The final fully connected layer was followed by a Softmax layer to output binary classification probabilities. The network was trained using the Adam optimizer with an initial learning rate of 0.001, a maximum of 50 epochs, and an early stopping strategy (training stopped if the validation loss did not improve for 5 consecutive epochs). Performance on the validation set was evaluated every 5 epochs. All input signals were normalized by Z-score (zero mean, unit variance) before being fed into the CNN. All signal processing and model training were conducted in the MATLAB R2023b environment. Five metrics were employed to comprehensively evaluate the classification performance from different dimensions. Accuracy reflects the overall correctness of the model’s classification across all samples. Precision measures the accuracy of positive defect predictions. Recall quantifies the proportion of true defects successfully detected. The F1 score is the harmonic mean of precision and recall. AUC characterizes the overall ability of the classifier to distinguish between defective and non-defective samples at different thresholds. In the context of roll defect detection, missed detections can lead to severe safety hazards; therefore, recall and AUC were identified as the two core metrics for evaluating preprocessing quality in this study. The former directly reflects the completeness of defect detection, while the latter comprehensively measures the separability of the preprocessed signals.

**Figure 12:**
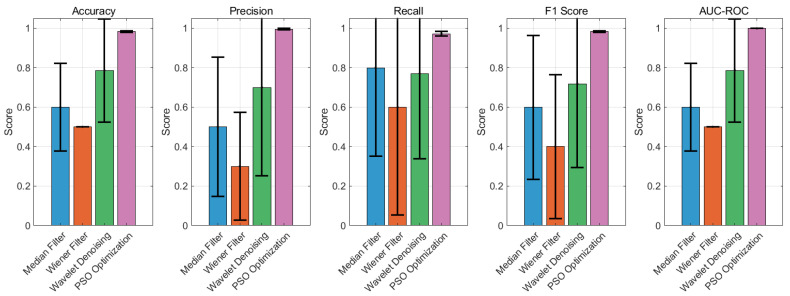
Bar chart of performance metrics for the four preprocessing methods.

Table 7 and Figure 12 present the classification performance comparison of the four preprocessing methods under 5-fold cross-validation. Overall, the proposed PSO-based method significantly outperformed the three traditional methods in accuracy (0.983 ± 0.01), precision (0.995 ± 0.01), recall (0.971 ± 0.01), F1-score (0.983 ± 0.01), and AUC-ROC (0.998 ± 0.01). Notably, the traditional methods (median filtering, Wiener filtering, wavelet denoising) exhibited large standard deviations across all metrics (e.g., recall of median filtering with a standard deviation of 0.45, precision of wavelet denoising with 0.447), whereas the proposed method achieved a standard deviation of only about 0.01 for every metric. This striking difference indicates that traditional methods are highly sensitive to complex background clutter, leading to drastic performance fluctuations across different data folds (they may fail completely in some folds while performing moderately in others), thus failing to guarantee stability and reliability for industrial inspection. In contrast, the proposed PSO method, incorporating an improved median filter and a PSO-optimized multi-stage FIR filter, adaptively suppresses time-varying clutter, producing preprocessed signals of consistently high quality. Consequently, it achieved a near-perfect classification performance with extremely low variance across all folds. Therefore, the proposed method not only delivers high accuracy but also exhibits exceptional robustness, making it a promising candidate for practical online industrial inspection.

**Figure 13:**
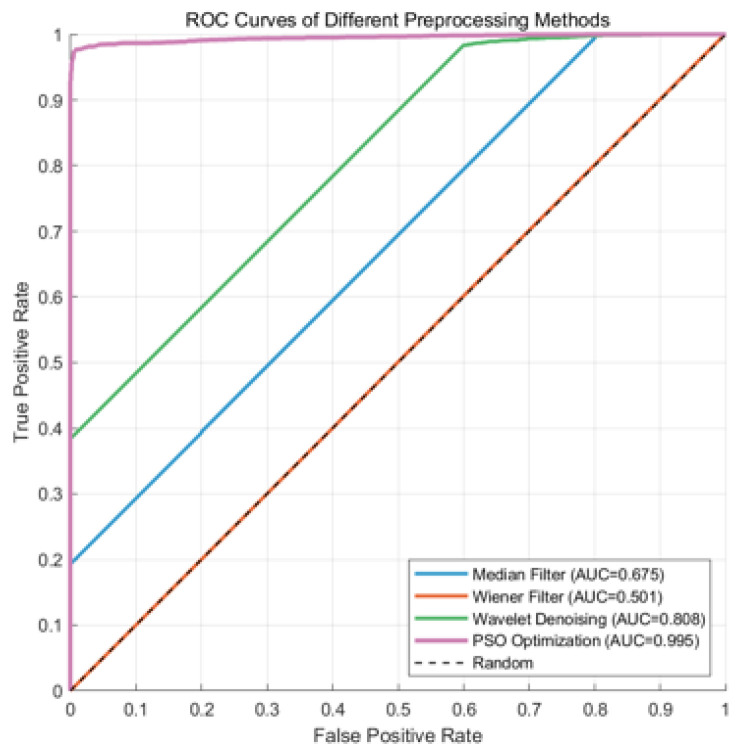
ROC curves of the four preprocessing methods.

Figure 13 shows the ROC curves and corresponding AUC values of the four preprocessing methods under fivefold cross-validation. The Wiener filter yielded an ROC curve close to the diagonal (AUC = 0.500), indicating that its classification performance is no better than random guessing. The median filter achieved an AUC of 0.679, slightly better than random. Wavelet denoising attained an AUC of 0.900, demonstrating good discriminative ability. In contrast, the proposed PSO-based method exhibited an ROC curve that was closest to the top-left corner, with an AUC as high as 0.996, which is nearly ideal. This confirms that the PSO method, by integrating an improved median filter and an adaptive FIR filter, effectively suppresses complex background clutter, enabling the CNN classifier to distinguish defective from non-defective signals with extremely high confidence, significantly outperforming traditional preprocessing methods.

### 4.5. Case Validation

**Figure 14:**
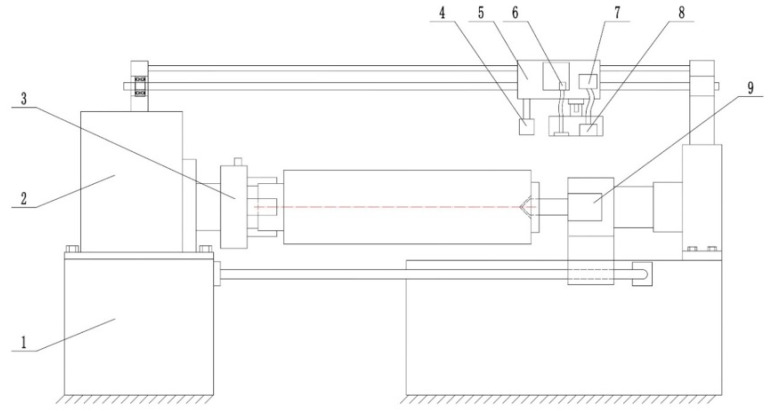
Schematic diagram of the experimental platform structure. 1—Base; 2—Motor and reducer; 3—Clamping device; 4—Displacement sensor; 5—Transverse and telescopic mechanism; 6—Couplant supply and recovery system; 7—Data acquisition and analysis module; 8—Ultrasonic probe; 9—Clamping mechanism.

To further validate the effectiveness and engineering feasibility of the proposed method in practical industrial scenarios, the preprocessing method was deployed on a roll ultrasonic online inspection system. The system was modified from a universal lathe (as shown in Figure 14) and equipped with a KY-CT350B digital ultrasonic flaw detector and a 2.5 MHz straight-beam probe. With the stepper motor-driven probe movement mechanism, it enables automated scanning over the entire length and full circumference of the roll.

**Figure 15:**
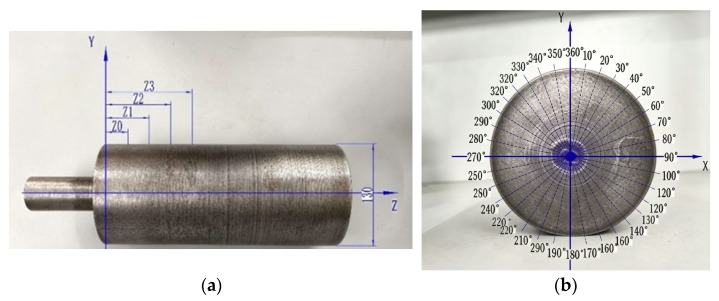
Schematic diagram of roll specimen inspection. (**a**) Schematic diagram of axial detection. (**b**) Schematic diagram of circumferential detection.

The inspection target was a #45 steel roll specimen with a diameter of 130 mm (as shown in Figure 15), containing a pre-fabricated cylindrical artificial defect (diameter 8 mm, length 30 mm, depth 10 mm to avoid near-surface coupling interference). During the inspection, the system first performed a coarse scan with a large step size to quickly locate suspected defect regions, followed by a fine scan with a reduced step size to acquire high-resolution echo data. The acquired signals were preprocessed by the proposed improved median filter (window radius = 12) and the PSO-optimized multi-stage FIR filter (order = 5), and then fed into the CNN classifier for automatic defect identification.

It should be noted that the signal model used in this system was identical to that used in the 5-fold cross-validation experiment in Section 4.3 (including time-varying clutter, spike noise, SNR of 10 dB, etc.). Therefore, the classification performance rigorously demonstrated in Section 4.3 directly reflects the actual detection capability of the proposed method. Specifically, with over 5000 samples (2500 defective, 2500 non-defective) in the 5-fold cross-validation, the proposed method achieved an accuracy of 0.983 ± 0.01, recall of 0.971 ± 0.01, precision of 0.995 ± 0.01, F1-score of 0.979 ± 0.01, and AUC-ROC of 0.998 ± 0.01, significantly outperforming traditional median filtering (accuracy 0.599 ± 0.22), Wiener filtering (0.500 ± 0.01), and wavelet denoising (0.785 ± 0.26). These results fully demonstrate the superiority of the proposed method in suppressing complex background clutter while preserving weak defect echoes.

Moreover, with the automated scanning procedure, the full-surface inspection of a single roll can be completed in about 5 min, representing an 83% improvement in inspection efficiency compared to manual inspection (approximately 30 min). These results indicate that the proposed method not only achieves high-precision defect identification but also meets the efficiency requirements of industrial online inspection, demonstrating its potential for transition from laboratory research to practical industrial applications.

## 5. Conclusions

This paper addresses the challenges of severe background clutter interference and the difficulty in extracting weak near-surface defect echoes in the ultrasonic testing of roll internal defects, and proposes a signal preprocessing method that integrates improved median filtering with a particle swarm optimization (PSO)-based multi-stage FIR filter. The method introduces a dynamic boundary outlier correction mechanism based on traditional median filtering to suppress spike pulses and coupling interference, and employs the PSO algorithm to adaptively determine the optimal weight vector of the multi-stage FIR filter, achieving the accurate estimation of background clutter and the reliable separation of defect signals. Experimental results demonstrate that the improved median filter achieved a favorable balance between noise suppression and signal fidelity at the optimal window radius, and the PSO algorithm outperformed both the genetic algorithm and the simulated annealing algorithm in terms of convergence speed and solution accuracy. Under the same CNN classifier, the proposed method surpassed median filtering, Wiener filtering, and wavelet denoising in key metrics including accuracy, precision, F1-score, and AUC, with the ROC curve closest to the upper-left corner, indicating markedly superior classification robustness. In practical industrial scenarios, the proposed method, combined with an automated scanning process, effectively reduces the inspection time for a single roll and significantly improves the defect detection rate compared with traditional ultrasonic testing, thereby validating its effectiveness and engineering feasibility under real-world operating conditions.

The current method is developed primarily for roll ultrasonic signals under specific inspection conditions. The filter order and PSO algorithm parameters still rely on experimental settings, and the adaptability to varying operating conditions remains to be enhanced. Future research can be pursued in the following directions: developing adaptive selection strategies for filter parameters to improve the generalization capability of the method to different roll materials, dimensions, and probe frequencies; exploring end-to-end lightweight deep learning denoising models to further reduce the dependence on manual feature extraction; extending the proposed method to the ultrasonic non-destructive testing of other large-scale metal components, such as rails and pipelines, to verify its generality and robustness; and accelerating the algorithm and deploying it on embedded platforms to meet the real-time online inspection requirements of industrial sites. Furthermore, to address the challenge of near-surface defect detection, dedicated investigations incorporating blind-zone deconvolution techniques can be conducted to further enhance the method’s capability for detecting tiny defects near the surface.

## Figures and Tables

**Table 1:** Comparison of experimental data for different window parameters.

Window Radius	SNR(dB)	RMSE	Window Width
7	7.8820	0.142980	15
8	8.4436	0.134028	17
9	8.8881	0.127341	19
10	9.2459	0.122202	21
11	9.6265	0.116963	23
12	9.9388	0.112832	25
13	10.2338	0.109065	27
14	10.5640	0.104996	29
15	10.8430	0.101677	31
16	11.0889	0.098840	33
17	11.2632	0.096876	35
18	11.4865	0.094417	37
19	11.5909	0.093289	39
20	11.7179	0.091935	41

**Table 2:** Performance increment data between adjacent window radii.

Window Radius Change	SNR Increment (dB)	RMSE Increment
7~8	+0.5616	−0.00895
8~9	+0.4445	−0.00669
9~10	+0.3578	−0.00514
10~11	+0.3805	−0.00524
11~12	+0.3123	−0.00413
12~13	+0.2950	−0.00377
13~14	+0.3302	−0.00407
14~15	+0.2790	−0.00332
15~16	+0.2458	−0.00284
16~17	+0.1743	−0.00196
17~18	+0.2233	−0.00246
18~19	+0.1044	−0.00113
19~20	+0.1270	−0.00135

**Table 3:** Objective function J and AIC for different filter orders.

d	J (Residual Sum of Squares)	AIC	J Decrease (%)
2	156.15	−17,323.29	-
3	153.81	−17,392.48	1.50%
4	151.63	−17,457.13	1.41%
5	149.38	−17,525.56	1.49%
6	148.41	−17,551.58	0.65%
7	146.48	−17,610.29	1.30%
8	144.55	−17,610.06	1.32%
9	143.98	−17,683.26	0.39%
10	142.89	−17,717.51	0.75%

**Table 4:** PSO algorithm parameter settings.

Parameter	Value	Parameter	Value
Population size	50	Number of iterations	60
Dimensionality	5	Inertia weight	0.8
Variable lower bounds	[−1,−1,−1,−1,−1]	Variable upper bounds	[1,1,1,1,1]
Individual learning factor	0.5	Global learning factor	0.6

**Table 5:** Parameter settings of PSO, GA and SA optimization algorithms.

Parameter	PSO	GA	SA
Population size	50	50	-
Iterations/generations/steps	60	60	3000
Total evaluations	3000	3000	3000
Learning factors	0.5/0.6	-	-
Crossover/mutation rate	-	0.85/0.15	-
Initial temperature/cooling rate	-	-	50/0.92

**Table 6:** Wilcoxon signed-rank test *p*-values.

Pair	*p*-Value
PSO vs. GA	7.56 $\times 10^{-10}$
PSO vs. SA	7.56 $\times 10^{-10}$

**Table 7:** Fivefold cross-validation results of the four preprocessing methods (mean ± SD).

Method	Accuracy	Precision	Recall	F1-Score	AUC
Median filtering	0.599 ± 0.22	0.500 ± 0.35	0.799 ± 0.45	0.599 ± 0.36	0.600 ± 0.22
Wiener filtering	0.500 ± 0.01	0.300 ± 0.27	0.600 ± 0.55	0.400 ± 0.37	0.500 ± 0.01
Wavelet denoising	0.785 ± 0.26	0.700 ± 0.447	0.770 ± 0.43	0.718 ± 0.42	0.786 ± 261
Proposed method	0.983 ± 0.01	0.995 ± 0.01	0.971 ± 0.01	0.983 ± 0.01	0.998 ± 0.01

## Data Availability

The data cannot be made publicly available upon publication due to the presence of sensitive personal information and confidential corporate details. Nevertheless, the data supporting the findings of this study are available upon reasonable request from the authors.
